# *FgVAC1* is an Essential Gene Required for Golgi-to-Vacuole Transport and Fungal Development in *Fusarium graminearum*

**DOI:** 10.1007/s12275-024-00160-x

**Published:** 2024-07-30

**Authors:** Sieun Kim, Jiyeun Park, You-Kyoung Han, Hokyoung Son

**Affiliations:** 1 Horticultural and Herbal Crop Environment Division, National Institute of Horticultural & Herbal Science, Rural Development Administration, Wanju, 55365 Republic of Korea; 2 Institute for Plant Sciences, University of Cologne, 50923 Cologne, Germany; 3 Department of Agricultural Biotechnology, Seoul National University, Seoul, 08826 Republic of Korea; 4 Research Institute of Agriculture and Life Sciences, Seoul National University, Seoul, 08826 Republic of Korea

**Keywords:** *Fusarium graminearum*, FgVac1, Vacuolar protein sorting, Endosome

## Abstract

*Fusarium graminearum* is an important plant pathogen that causes head blight in cereal crops such as wheat, barley, and rice worldwide. In this study, we identified and functionally characterized *FgVAC1*, an essential gene in *F. graminearum* that encodes a Rab5 effector involved in membrane tethering functions. The essentiality of *FgVAC1* was confirmed through a conditional promoter replacement strategy using the zearalenone-inducible promoter (*P*_*ZEAR*_). Cytological analyses revealed that FgVac1 colocalizes with FgRab51 on early endosomes and regulates the proper transport of the vacuolar hydrolase FgCpy1 to the vacuole. Suppression of *FgVAC1* led to inhibited vegetative growth, reduced asexual and sexual reproduction, decreased deoxynivalenol (DON) biosynthesis, and diminished pathogenicity. Our findings highlight the significant role of FgVac1 in vacuolar protein sorting, fungal development, and plant infection in *F. graminearum*.

**Supplementary Information:**

The online version contains supplementary material available at 10.1007/s12275-024-00160-x.

## Introduction

*Fusarium graminearum* is a major pathogen responsible for Fusarium head blight (FHB) in cereal crops and ear rot in maize (Leslie & Summerell, 2006). This fungus not only causes substantial yield losses but also produces mycotoxins such as deoxynivalenol (DON) and zearalenone (ZEA), which are hazardous to human and animal health (Goswami & Kistler, 2004). Currently, the primary strategy for controlling FHB involves using sterol demethylation inhibitors (DMIs) (McMullen et al., 2012). However, the extensive use of DMI fungicides has led to reduce sensitivity in *F. graminearum* (Spolti et al., 2013), emphasizing the need for new disease control targets. Essential genes, which are crucial for an organism's survival, are prime targets for antifungal drugs. Recently, we identified 13 essential genes in *F. graminearum* as potential targets for disease control (Kim et al., 2023a, 2023b). Among these, the *Fg04221* gene is homologous to the *S. cerevisiae VAC1/VPS19/PEP7* gene, which is involved in vesicle-mediated vacuolar protein sorting.

Secretory and vacuolar proteins transit through the Golgi and the *trans*-Golgi network (TGN) on their way to final destinations, such as the plasma membrane and the vacuole (Shimizu & Uemura, 2022; Shimizu et al., 2021). Vacuolar proteins either pass through the prevacuolar compartment (PVC) (e.g., carboxypeptidase Y (CPY)) or go directly to the vacuole (e.g., alkaline phosphatase (ALP)) (Feyder et al., 2015). Specific mediators, including tethering factors, SNAREs, Rab GTPases, guanine-nucleotide exchange factors (GEFs), and Sec1/Munc18 (SM) proteins, regulate vesicle fusion events (Caza et al., 2018). Cargo-loaded vesicles from the Golgi fuse with the early endosomes, which mature into late endosomes as Rab5s are replaced by Rab7 (Pinar & Peñalva, 2021). These cargoes are either sent to the vacuole or recycled back to the TGN (Caza et al., 2018).

Vac1/Vps19/Pep7, a member of the class D Vps group, is a tethering protein that facilitates the targeting of Golgi-derived vesicles to early endosomes in collaboration with the SM protein Vps45 and the SNARE Pep12 (Pinar & Peñalva, 2021). This protein contains a phosphatidylinositol 3-phosphate (PI3P)-binding FYVE domain and a Rab-binding domain, allowing its recruitment to early endosomes through the detection of Rab5-GTP and PI3P, the product of phosphatidylinositol 3-kinase (PI3K) Vps34 (Pinar & Peñalva, 2021).

In *S. cerevisiae*, the *vac1* mutant was found to be defective in the vacuolar segregation process (Weisman & Wickner, 1992). Loss of Pep12, Vps21, or Vps45 in the Class D Vps group resulted in the accumulation of transport vesicles in the cytosol, suggesting their roles in TGN to PVC transport (Gerrard et al., 2000). Additionally, *vps* deletion mutants in yeast exhibited increased sensitivity to zinc (Zhao et al., 2020). In *C. albicans,* the *vac1* mutant displayed defective endosomal vesicle transport and highly fragmented vacuoles (Veses et al., 2009). Vac1 also contributed to resistance against metal ions and affected several virulence factors in* C. albicans* (Franke et al., 2006). In *F. graminearum*, several *VPS* genes have been found to play important roles in vegetative growth, asexual or sexual development, DON production, and pathogenicity by mediating vesicle trafficking and vacuole fusion (Abubakar et al., 2021; Li et al., 2017, 2019; Xie et al., 2019; Yang et al., 2020). However, existing studies predominantly focus on Rab proteins, SNAREs, or the ESCRT (Endosomal sorting complexes required for transport) machinery, while the Golgi-to-endosomal compartments, particularly the role of the tethering protein Vac1, remains inadequately characterized in plant pathogenic fungi.

This study aimed to elucidate the role of FgVac1 in *F. graminearum*. Our data showed that FgVac1 is required for vegetative growth, asexual and sexual development, vacuolar protein sorting, DON production, and pathogenicity in *F. graminearum*. Localization studies revealed that FgVac1 is localized to early endosomes. Suppression of *FgVAC1* led to the mislocalization of FgCpy1, a vacuolar enzyme, and increased sensitivity to metal ions. These results underscore the critical role of FgVac1 in diverse biological processes, making it a promising target for the control and management of FHB.

## Materials and Methods

### Strains and Culture Conditions

The wild-type strain Z-3639 of *F. graminearum* (Bowden & Leslie, 1999) was used as the host strain for transformation experiments. All strains (Table S1) were maintained as mycelial suspensions in 20% glycerol solution at − 80 °C. Culture media were prepared according to procedures described in the *Fusarium* laboratory manual (Leslie & Summerell, 2006). Complete medium (CM) and minimal medium (MM) were used to assess the mycelial growth of *F. graminearum* strains. Conidial production was induced using carboxymethyl cellulose (CMC) medium or yeast malt agar (YMA) (Cappellini & Peterson, 1965; Harris, 2005).

### Nucleic Acid Manipulations, Southern Blotting, and Quantitative Reverse Transcription (qRT)-PCR

Fungal genomic DNA was extracted from freeze-dried mycelial powder using cetyl-trimethylammonium-based (CTAB) method (Leslie & Summerell, 2006). Southern blot hybridization was conducted using the North2South™ Biotin Random Prime Labeling Kit and North2South™ Chemiluminescent Hybridization and Detection Kit (Thermo Fisher Scientific). PCR primers listed in Table S2 were synthesized by an oligonucleotide synthesis facility (Bioneer).

For quantitative reverse transcription (qRT)-PCR**, t**otal RNA was isolated using the Easy-Spin Total RNA Extraction Kit (Intron Biotech). First- strand cDNA synthesis was performed with SuperScript III reverse transcriptase from Invitrogen. The qRT-PCR was carried out using SYBR Green Supermix (Bio-Rad) on the Bio-Rad CFX Real-Time PCR System. The endogenous cyclophilin gene (*CYP1*; FGSG_07439) was used as a reference gene. Each PCR assay was repeated three times with three biological replicates. The relative transcript levels of the target genes were calculated using the 2^–ΔΔCt^ method.

### Genetic Manipulations and Fungal Transformations

To generate *P*_*ZEAR*_-*FgVAC1* mutants, the *P*_*ZEAR*_-*FgVAC1* fusion construct was generated using the yeast gap repair approach (Bruno et al., 2004). The *P*_*ZEAR*_ promoter and the open reading frame (ORF) of *FgVAC1* were amplified from the genomic DNA of the wild-type strain. The resulting amplicons were fused and co-transformed with Xho1-digested pDL2 into the yeast strain PJ69-4A (Zhou et al., 2011) using the Alkali-Cation Yeast Transformation Kit (MP Bio). The *P*_*ZEAR*_-*FgVAC1* fusion vector obtained from the yeast transformants was transformed into *Escherichia coli* DH10B. After verification by sequencing, plasmid DNA was extracted with the DNA-spin Plasmid DNA Purification Kit (Intron Biotech). The p*P*_*ZEAR*_-*FgVAC1* plasmid, containing the *P*_*ZEAR*_-*FgVAC1* construct, was introduced into the fungal wild-type strain. Transformation experiments for *F. graminearum* were performed as described in the previous study (Son et al., 2011).

To delete the native *FgVAC1* gene in the resulting transformants, we constructed fusion PCR products using the double-joint (DJ) PCR method (Yu et al., 2004). The 5’ and 3’ flanking regions of *FgVAC1* were amplified from the genomic DNA of the wild-type strain, and the geneticin resistance cassette (*GEN*) was amplified from the pII99 plasmid (Namiki et al., 2001). These three fragments were fused using the DJ PCR method, and the final products were generated using nested primers. The resulting PCR products were transformed into fungal protoplasts expressing the p*P*_*ZEAR*_-*FgVAC1* plasmid. Transformants were screened based on their mycelial growth on complete medium under repressing conditions and CM supplemented with β-estradiol under inducing conditions. The mutants were confirmed through Southern blot analysis using a flanking region probe.

To generate the FgVac1-GFP strain, the *RP27-FgVAC1-GFP* fusion construct was created using the yeast gap repair approach (Bruno et al., 2004). The ORF of *FgVAC1* was amplified from the genomic DNA of the wild-type strain, and co-transformed with Xho1-digested pDL2 into the yeast strain PJ69-4A, as described above. The *RP27-FgVAC1-GFP* fusion vector, which carries the *Magnaporthe oryzae* ribosomal protein 27 promoter (RP27), was recovered from the yeast transformants. The subsequent process was carried out as described above. The verified vectors were transformed into the wild-type strain.

For the construction of the FgCpy1-green fluorescence protein (GFP) strain, the ORF of *FgCPY1* along with its native promoter was amplified from the genomic DNA of the wild-type strain. The resulting construct was co-transformed with Xho1-digested pDL2 into the yeast strain PJ69-4A. The subsequent steps followed the same procedure as outlined above.

 To generate red fluorescence protein (RFP)-FgRab51 strain, the ORF of *FgRAB51* and its native promoter were amplified from the fungal genomic DNA, and the RFP sequence was amplified from the pLC25 plasmid. These three amplicons were fused using the DJ PCR method (Yu et al., 2004), and the final constructs were obtained using the nested primers. The resulting construct and Xho1-digested pDL2 were co-transformed into the yeast strain PJ69-4A, following the same procedure. The RFP-FgRab7 strain was constructed via the same strategy.

### Conidiation, Sexual Development and Genetic Crosses

For conidiation assays, fresh mycelial plugs from CM were inoculated in 5 ml of CMC medium and incubated for 4 days on a rotary shaker set at 200 rpm. Conidia were quantified using a hemacytometer. To induce conidiation in the *P*_*ZEAR*_-*FgVAC1* mutant, 10 μM β-estradiol was added to the CMC medium.

For sexual development assays, fungal strains were cultured on carrot agar for 5 days, after which aerial mycelia were removed with 0.4 ml of a sterile 2.5% Tween 60 solution (Leslie & Summerell, 2006). The plates were subsequently incubated under near-UV light (wavelength: 365 nm; Sankyo Denki). The number and maturation of perithecia were assessed after 7–9 days. To induce perithecial formation in the *P*_*ZEAR*_-*FgVAC1* mutant, 30 μM β-estradiol was added to the carrot agar daily post-induction. In outcrossing experiments, the female strain was fertilized with 1 ml of conidial suspension from the male strain 5 days after initial inoculation on carrot agar.

### Microscopic Observation

For fluorescence imaging of GFP and RFP signals in the FgVac1-GFP;RFP-FgRab7 and FgVac1-GFP;RFP-FgRab51 strains, conidial suspensions were inoculated in CM at a concentration of 2 × 10^5^ conidia/ml, and cultured for 24 h on a rotary shaker (200 rpm). For CMAC (7-amino-4-chloromethylcoumarin) and calcofluor white (CFW) staining, fresh mycelia collected after 24 h of incubation were stained with 250 μM CMAC and 10 μg/ml CFW, respectively. Samples were examined for CMAC and CFW staining signals with a Leica DM6 B microscope (Leica Microsystems) equipped with a Leica DMC6200 camera and suitable fluorescent filters. The perithecia were imaged 8 days after induction of sexual development using a Zeiss SteREO Lumar.V12 microscope (Carl Zeiss).

### Mycotoxin Analysis and Virulence Test

To assess trichothecene production, a conidial suspension (1 × 10^4^ conidia/ml) was inoculated into a defined medium supplemented with 5 mM agmatine (MMA) and cultured for 6 days at 25 °C under stationary conditions (Gardiner et al., 2009). For induction of trichothecene production in the *P*_*ZEAR*_-*FgVAC1* mutant, 20 μM β-estradiol was added to the MMA medium. The MMA cultures were extracted using an ethyl acetate–methanol mixture (4:1, v/v). The resulting extracts were dried and dissolved in a mobile phase (10% acetonitrile solution) for analysis by high-performance liquid chromatography (HPLC) (Kim et al., 2023a, 2023b). A Shimadzu Prominence system equipped with a C18 column was employed for chromatographic separation The total amount of deoxynivalenol (DON) was quantified relative to the biomass produced by each strain in MMA. Detection was performed using diode-array detection, specifically targeting DON at a wavelength of 235 nm.

For the assessment of fungal virulence, a conidial suspension (1 × 10^7^ conidia/ml) was inoculated onto cut wheat coleoptiles (cultivar: Eunpamil) (Jia et al., 2017). The inoculated wheat coleoptiles were then placed in a humid chamber, and the lesion sizes were measured 7 days after inoculation. To induce disease development in the *P*_*ZEAR*_-*FgVAC1* mutant, 20 μM β-estradiol was applied daily to the inoculated spots.

## Results

### Identification of FgVac1 in *F. graminearum*

**Fig. 1:**
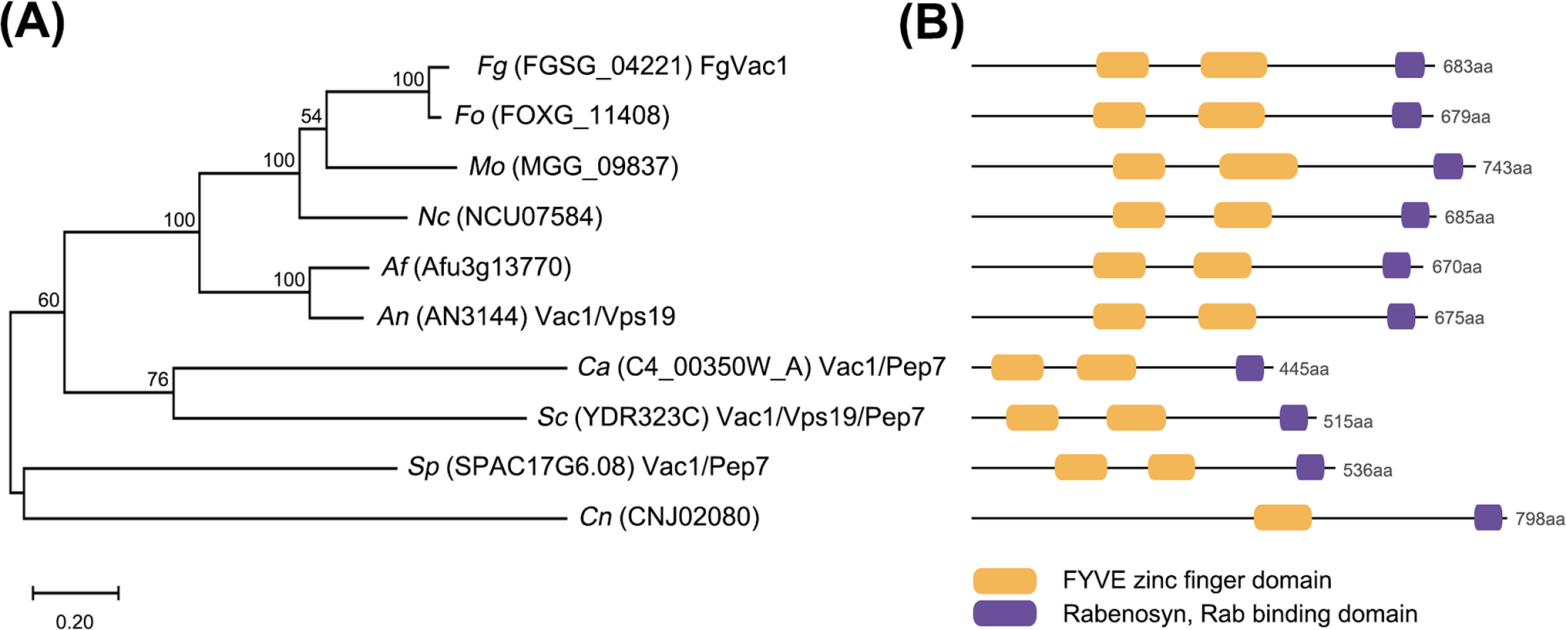
Phylogenetic tree and domain architecture of FgVac1 in *F. graminearum*. **A** A phylogenetic tree based on amino acid sequence alignments of selected Vac1/Vps19/Pep7 homologs in *F. graminearum* and other fungi. The MEGA-X program was used to perform ClustalW alignment using the neighbor-joining method. Numbers at nodes represent bootstrap values for 1000 replicates. *Fg* *Fusarium graminearum*, *Fo* *Fusarium oxysporum*, *Mo* *Magnaporthe oryzae,* *Nc* *Neurospora crassa,* *Af* *Aspergillus fumigatus*, *An* *Aspergillus nidulans,* *Ca* *Candida albicans*, *Sc* *Saccharomyces cerevisiae*, *Sp* *Schizosaccharomyces pombe*, *Cn* *Cryptococcus neoformans.* **B** Domain architectures of selected Vac1/Vps19/Pep7 homologs in *F. graminearum* and other fungi. Conserved domains are represented by color boxes

Our previous study showed that FGSG_04221 is an essential gene in *F. graminearum* (Kim et al., 2023a, 2023b). Using BLASTp analysis in the Saccharomyces Genome Database (https://www.yeastgenom-e.org), we identified the nearest homologue of FGSG_04221 as *S. cerevisiae* Vac1/Vps19/Pep7, which is involved in vesicle-mediated vacuolar protein sorting. FGSG_04221 is predicted to encode a 683-amino acid (aa) polypeptide. Phylogenetic analysis showed that FGSG_04221 is well conserved with a high amino acid sequence identity to Pep7/Vac1/Vps19 proteins in other fungi (Fig. 1A). It shares 93% amino acid similarity to *Fusarium oxysporum* FOXG_11408, 58% to *Magnaporthe* *oryzae* MGG_09837, 59% to *Neurospora crassa* NCU07584, 49% to *Aspergillus fumigatus* Afu3g13770, 48% to *Aspergillus nidulans* Vac1/Vps19, 20% to *C. albicans* Vac1/Pep7, 25% to *S. cerevisiae* Vac1/Vps19/Pep7, 26% to *Schizosaccharomyces pombe* Vac1/Pep7, and 22% to *Cryptococcus neoformans* CNJ02080.

Domain prediction indicates that FGSG_04221 contains a FYVE zinc finger domain (180–256 aa and 333–429 aa) and Rabenosyn (Rab binding) domain (622–663 aa), similar to other Vac1/Vps19/Pep7 proteins (Fig. 1B). Given the strong similarity among Vac1/Vps19/Pep7 orthologs, we designated FGSG_04221 as FgVac1.

### FgVac1 is Required for Vegetative Growth, Conidiation, and Sexual Development

**Fig. 2:**
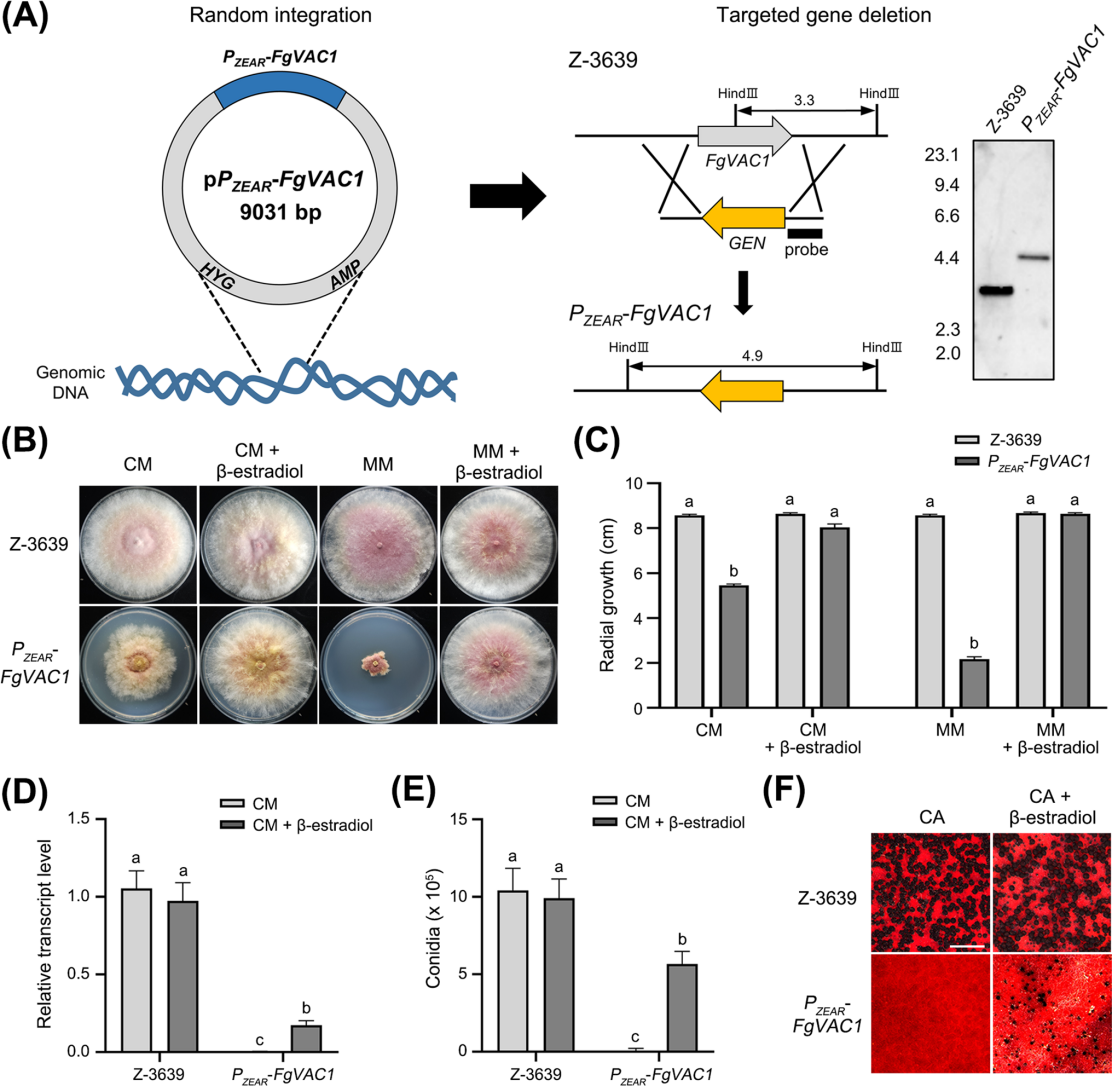
Phenotypes of the *P*_*ZEAR*_–*FgVAC1* mutant in vegetative growth, conidiation, and sexual reproduction. **A** Schematic representation for the CPR strategy used to generate the* P*_*ZEAR*_–*FgVAC1* mutant. The p*P*_*ZEAR*_-*FgVAC1* plasmid, harboring the *P*_*ZEAR*_-*FgVAC1* construct, was introduced into the *F. graminearum* wild-type strain Z-3639 (left panel), followed by deletion of the native *FgVAC1* gene via homologous recombination (right panel). Gene deletion was confirmed by Southern blot analysis, with DNA standard sizes (kb) indicated on the left of the blot. **B** Mycelial growth of the wild-type Z-3639 and the *P*_*ZEAR*_-*FgVAC1* mutant under repressing conditions (CM and MM) and inducing conditions (medium supplemented with 30 μM β-estradiol). The photographs were taken 4 days after inoculation. **C** Colony diameters of *F. graminearum* strains. Colony diameters were measured 4 days after inoculation, with three biological replicates for each condition. **D** Relative transcript levels of *FgVAC1* in *F. graminearum* strains. Fresh mycelia of *F. graminearum* strains were cultured in CM and CM supplemented with 30 μM β-estradiol for 1 h, and the relative transcript levels of *FgVAC1* were analyzed by qRT-PCR. **E** Conidial production. Conidial production was assessed by incubating *F. graminearum* strains in CMC and CMC supplemented with 10 μM β-estradiol. The number of conidia produced by each strain was measured 4 days after inoculation. **F** Sexual development of *F. graminearum* strains on carrot agar (CA). The photographs were taken 9 days after sexual induction, with 30 μM β-estradiol supplemented daily post-induction. Scale bar = 1000 μm. **C**–**E** All data were statistically analyzed using one-way ANOVA, followed by Tukey’s multiple comparison test

To characterize the role of FgVac1 in fungal development, we generated the conditional promoter replacement (CPR) mutant using the zearalenone-inducible promoter, *P*_*ZEAR*_, which is activated by zearalenone or the estrogenic compound β-estradiol (Lee et al., 2010). The p*P*_*ZEAR*_-*FgVAC1* plasmid containing the *P*_*ZEAR*_-*FgVAC1* construct was introduced into the wild-type strain of *F. graminearum*, followed by deletion of the native gene through homologous recombination and a split-marker method (Fig. 2A). Gene deletion was confirmed using Southern blot analysis. The *P*_*ZEAR*_-*FgVAC1* mutant was validated by assessing its mycelial growth (Fig. 2B, C) and relative transcript levels through qRT-PCR analysis (Fig. 2D) under both repressing conditions (non-supplemented medium) and inducing conditions (medium supplemented with 30 μM β-estradiol).

We initially evaluated the vegetative growth of the *P*_*ZEAR*_-*FgVAC1* mutant on CM and MM agar plates (Fig. 2B, C). The *P*_*ZEAR*_-*FgVAC1* mutant exhibited severe growth inhibition, with growth reduced to 36.7% of the wild-type strain in CM and 74.8% in MM. This growth inhibition was effectively restored by supplementation with β-estradiol. We further examined the ability of the *P*_*ZEAR*_-*FgVAC1* mutant to produce conidia and found that the mutant rarely produced conidia in CMC medium (Fig. 2E). Conidial production of the *P*_*ZEAR*_-*FgVAC1* mutant was recovered to 54% of the wild-type level with β-estradiol supplementation.

Given the importance of sexual reproduction in FHB epidemics, we assessed sexual reproduction of the *P*_*ZEAR*_-*FgVAC1* mutant (Fig. 2F). The *P*_*ZEAR*_-*FgVAC1* mutant failed to produce any perithecia, whereas the wile type produced abundant perithecia on carrot agar plates. Although β-estradiol supplementation induced smaller immature perithecia in the *P*_*ZEAR*_-*FgVAC1* mutant, none of them developed ascospores. Collectively, these results suggest that *FgVAC1* is required for vegetative growth, asexual and sexual reproduction in *F. graminearum*.

### FgVac1 Localizes to Early Endosomes in *F. graminearum*

**Fig. 3:**
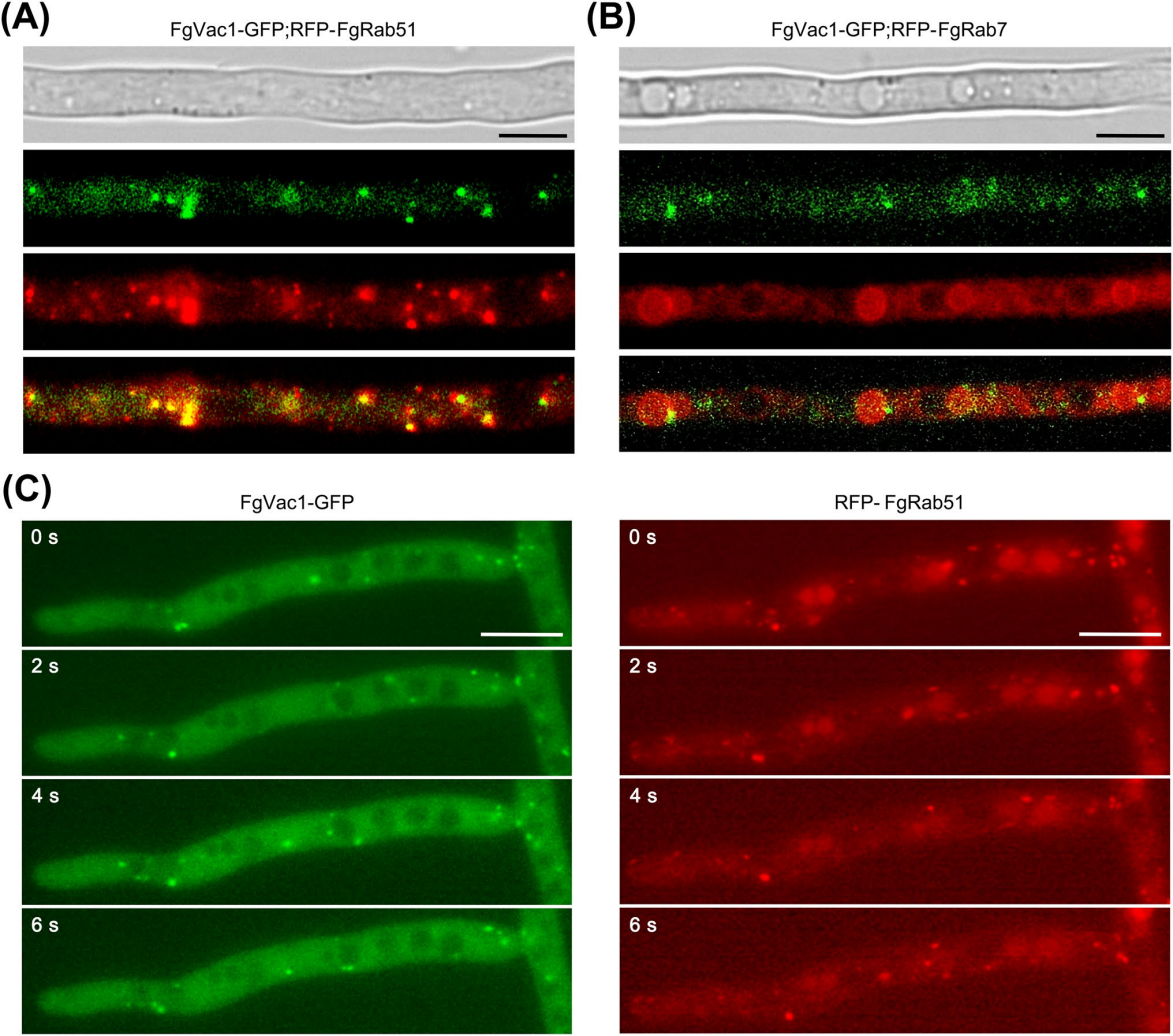
Subcellular localization of FgVac1-GFP in growing hyphae of *F. graminearum*. **A**, **B** Colocalization analyses were performed using strains co-expressing FgVac1-GFP with RFP-FgRab51 (early endosome marker) (**A**) and RFP-FgRab7 (late endosomal or vacuolar membrane marker) (**B**). Scale bar = 20 μm. **C** Time-lapse imaging of FgVac1-GFP and RFP-FgRab51 dynamics. Frames are taken every 2 s. Scale bar = 20 μm

In yeast, Vac1 localizes to early endosomes, where it interacts with the SM (Sec1/Munc-18) protein Vps45 and the SNARE protein Pep12, facilitating the endosomal docking of Golgi-derived vesicles (Peterson et al., 1999). To investigate the role of FgVac1, we fused GFP to its C-terminus and examined the subcellular localization. The FgVac1-GFP signals were observed in punctate structures throughout the cell (Fig. 3).

In *F. graminearum*, FgRab51 serves as early endosome marker protein, while FgRab7 is used as a marker for late endosomes and vacuolar membranes (Li et al., 2019; Zheng et al., 2015). To determine whether FgVac1 localizes to early or late endosomes, we utilized RFP tags to create RFP-FgRab51 and RFP-FgRab7 constructs. RFP-FgRab51 and RFP-FgRab7 strains were constructed and subsequently crossed with the FgVac1-GFP expressing strain for co-localization analysis. In the resulting transformants, the FgVac1-GFP signal significantly overlapped with RFP-FgRab51 in punctate structures within vegetative hyphae (Fig. 3A). FgVac1 exhibited colocalization with FgRab7 in only a few instances (Fig. 3B). Notably, FgVac1-GFP in punctate structures exhibited characteristics of being short-range, fast-moving, and highly dynamic, similar to RFP-FgRab51 (Fig. 3C, Videos S1 and S2). Taken together, these results indicate that FgVac1 predominantly localized to the early endosomes with endosomal vesicle trafficking in *F. graminearum*.

### FgVac1 is Essential for Vacuolar Protein Sorting

**Fig. 4:**
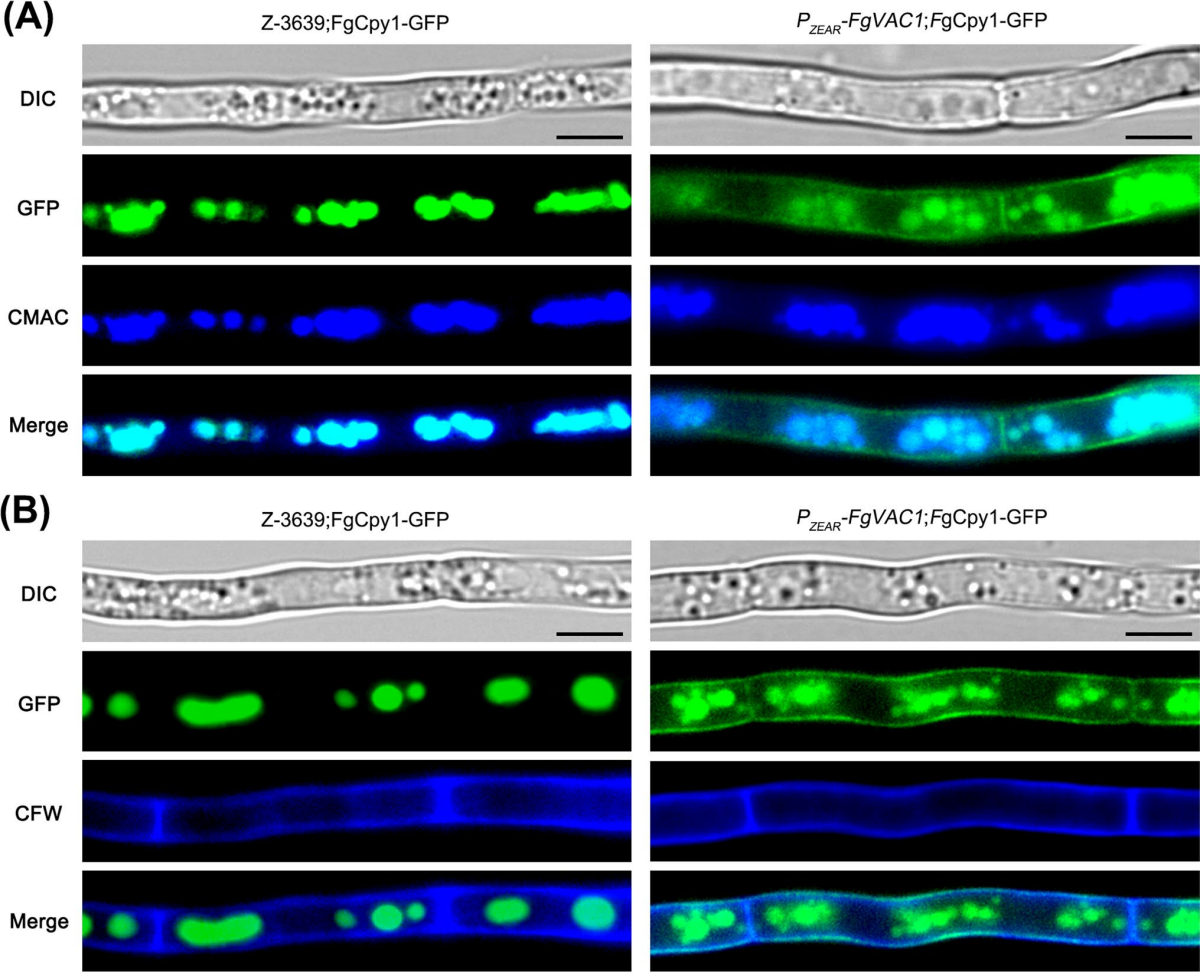
Subcellular localization of FgCpy1-GFP in *F. graminearum* strains. **A**, **B** FgCpy1 was fused with GFP in both the wild-type Z-3639 and the* P*_*ZEAR*_-*FgVAC1* mutant. The mycelia of these strains were stained with CMAC (**A**) to visualize vacuoles or with calcofluor white (CFW) (**B**) to stain the fungal cell wall. Scale bar = 20 μm

To explore the role of FgVac1 in vacuolar protein sorting in *F. graminearum*, we investigated the cellular localization of FgCpy1 in the *P*_*ZEAR*_-*FgVAC1* mutant and the wild-type strain (Fig. 4). When the vacuolar protein sorting system is impaired, Cpy1 is missorted and secreted into the external medium, thereby localizing on the cell wall (Yoon et al., 2010). A FgCpy1-GFP construct was created for microscopic analysis and introduced into both strains. Localization patterns of FgCpy1-GFP were determined using the vacuolar marker CMAC and the cell wall dye CFW. In the wild-type strain, the FgCpy1-GFP signals were primarily localized within vacuoles, as confirmed by their colocalization with CMAC staining, and no GFP signals were observed in septa or the cell wall. In contrast, in the *P*_*ZEAR*_-*FgVAC1* mutant, FgCpy1-GFP signals were detected in both vacuoles and the cell wall, showing colocalization with both CMAC and CFW staining. These observations suggest that impaired function of FgVac1 resulted in mislocalization of the FgCpy1 protein, diverting it away from its typical pathway to the vacuole and potentially directing it towards the secretory pathway.

### FgVac1 Plays a Crucial Role in Regulating Responses to Metal Ion Stress

**Fig. 5:**
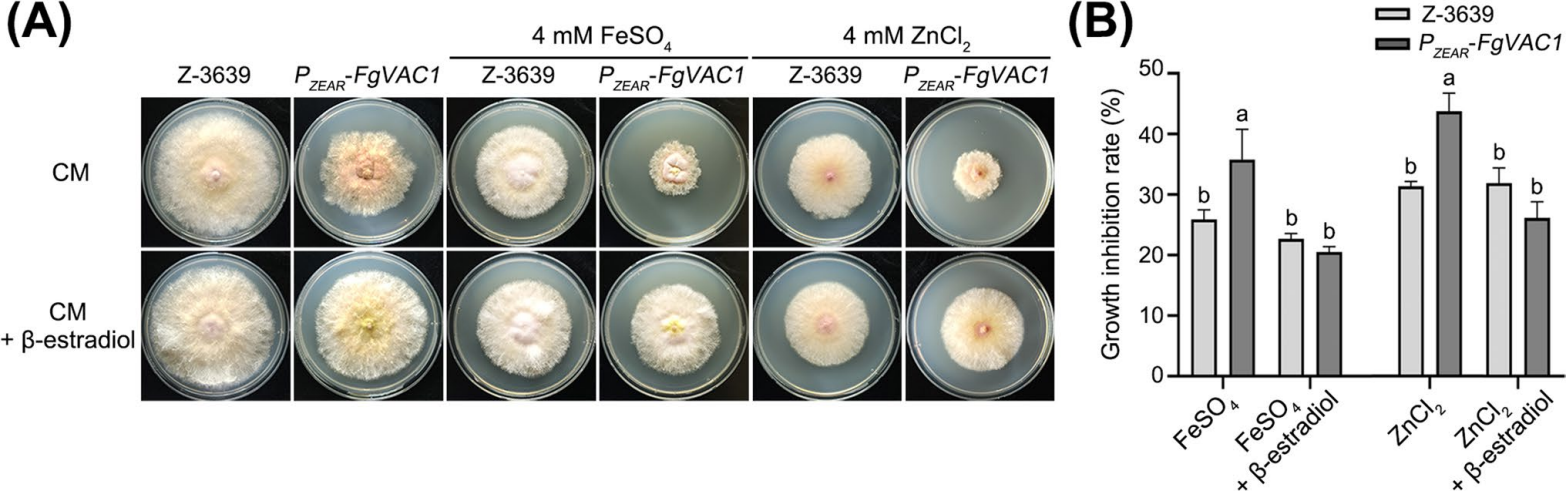
Metal ion sensitivity of *F. graminearum* strains. **A** The strains were inoculated on CM and CM supplemented with 4 mM FeSO_4_ or 4 mM ZnCl_2_, with or without 30 μM β-estradiol. The pictures were taken 4 days after inoculation. **B** Growth inhibition rates of *F. graminearum* strains on metal ion-supplemented media. Different letters indicate a significant difference (*P* < 0.05) based on one-way ANOVA followed by Tukey’s multiple comparison test

The vacuole is an important subcellular site for metal ion detoxification. In yeast, the *vac1* null mutant showed increased sensitivity to metal ions, indicating that Vac1 is involved in metal ion resistance (Franke et al., 2006; Pagani et al., 2007). To assess whether FgVac1 contributes to metal ion resistance in *F. graminearum*, both the wild-type strain and the *P*_*ZEAR*_-*FgVAC1* mutant were cultured on CM supplemented with 4 mM FeSO_4_ or 4 mM ZnCl_2_. As shown in Fig. 5A, the *P*_*ZEAR*_-*FgVAC1* mutant displayed greater sensitivity to iron and zinc compared to the wild-type strain. The wild-type strain showed growth inhibition rates of 25.9% with iron and 30.5% with zinc, whereas the *P*_*ZEAR*_-*FgVAC1* mutant exhibited growth inhibition rates of 36.4% with iron and 43.7% with zinc, respectively (Fig. 5B). Supplementation with β-estradiol restored the growth inhibition rate of the *P*_*ZEAR*_-*FgVAC1* mutant to wild-type levels. These findings suggest that FgVac1 is involved in metal ion resistance in *F. graminearum*.

### FgVac1 is Required for Trichothecene Production and Virulence *in F. graminearum*

**Fig. 6:**
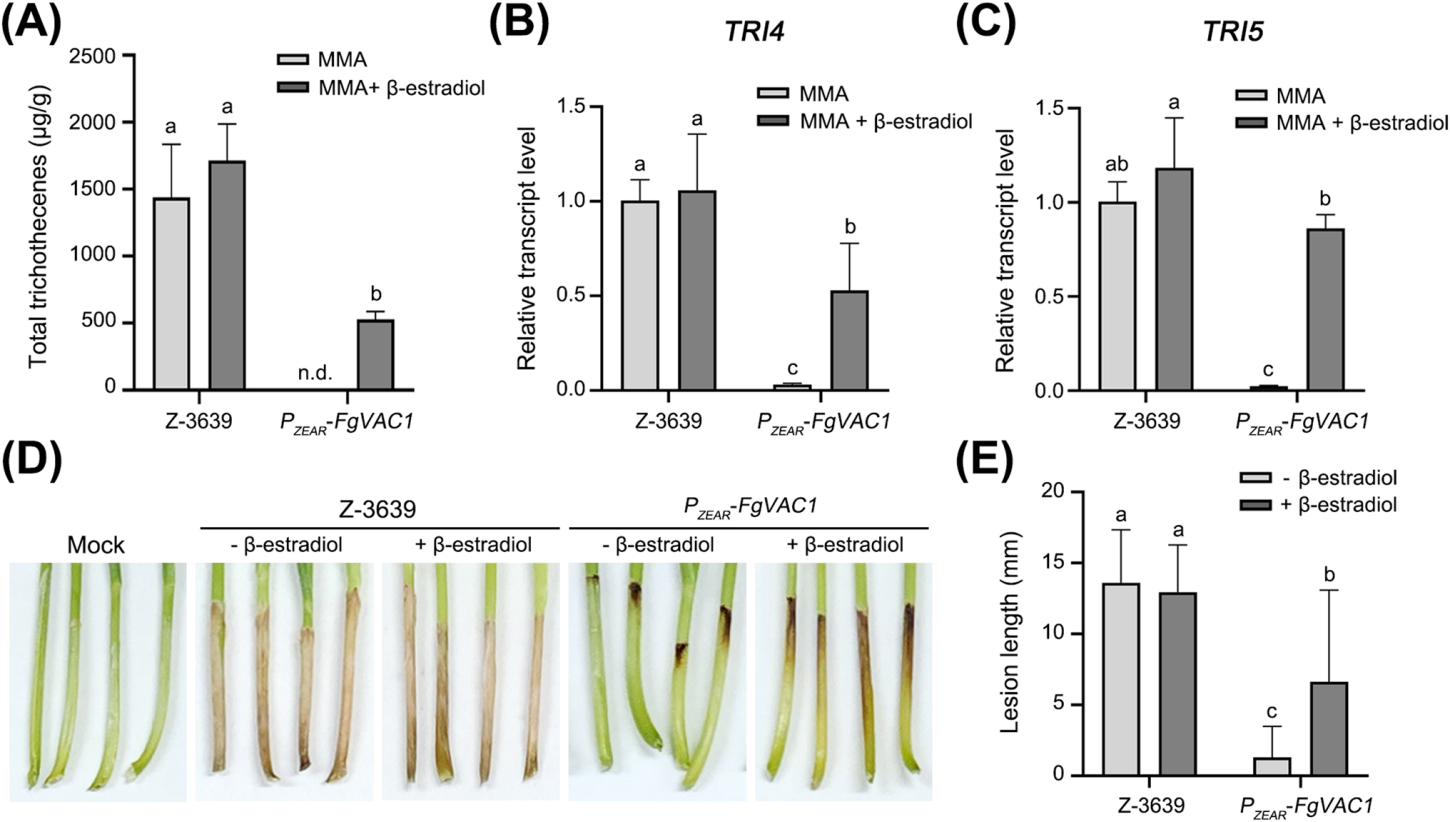
DON production and virulence assay. **A** DON production normalized to fungal mass. The total amount of DON was analyzed in *F. graminearum* strains 6 days after inoculation in MMA. *n.d.* not detected. **B**, **C** Relative transcript levels of *TRI* genes. Relative transcript levels of *TRI4* (**B**) and *TRI5* (**C**) in 4-day-old MMA cultures of *F. graminearum* strains were analyzed via qRT-PCR. **D** Disease symptoms in wheat coleoptiles. The apices of wheat coleoptiles were excised and inoculated with conidial suspensions of *F. graminearum* strains. The photographs were taken 7 days after inoculation, with daily supplementation of 30 μM β-estradiol post-induction. **E** Lesion lengths on wheat coleoptiles infected with the indicated strains. Lesion lengths were measured 7 days after inoculation. Fifteen biological replicates were used for each condition. (**A**–**C**, **E**) All experimental data were subjected to statistical analysis using one-way ANOVA, followed by Tukey’s multiple comparison test

To investigate the role of FgVac1 in the pathogenicity of *F. graminearum*, we examined the DON production*,* and assessed lesion sizes on wheat coleoptiles. DON production was measured in strains cultured in MMA and normalized to fungal mass (Fig. 6A). DON was not detected in the *P*_*ZEAR*_-*FgVAC1* mutant, while supplementation with β-estradiol restored DON production to 36.6% of the wild-type level. Relative transcription levels of the trichothecene biosynthesis genes *TRI4* and *TRI5* were significantly reduced in the *P*_*ZEAR*_-*FgVAC1* mutant compared to the wild-type (Fig. 6B and 6C). β-estradiol supplementation significantly restored the relative transcript levels of *TRI4* and *TRI5*, indicating that FgVac1 participates in DON production by regulating the expression of trichothecene biosynthesis genes in *F. graminearum*.

We further explored the role of FgVac1 in plant infection by inoculating wheat coleoptiles with conidial suspensions of the wild-type and the *P*_*ZEAR*_-*FgVAC1* mutant. At 7 days post-inoculation, the wild-type caused typical necrotic lesions, whereas the *P*_*ZEAR*_-*FgVAC1* mutant caused only mild lesions, approximately 10% of the wild-type level. β-estradiol supplementation restored lesion size of the mutant to 48.8% of the lesion size caused by the wild-type strain. These findings demonstrate that FgVac1 plays a critical role in vacuolar protein sorting process, fungal development, metal ion resistance, DON production, and virulence in *F. graminearum.*

## Discussion

Vac1/Vps19/Pep7 plays a pivotal role in the vesicle-mediated vacuolar protein sorting system, essential for directing Golgi-derived vesicles to vacuoles. Despite the well-documented functions of Vps proteins, the physiological and pathological roles of FgVac1 in plant pathogenic fungi, have been largely unexplored. This study identified FgVac1 as a crucial component involved in vacuolar protein sorting, impacting key biological processes such as vegetative growth, reproduction, DON production, and virulence.

In *F. graminearum*, genes involved in vacuolar protein sorting, including *VPS9, VPS27, VPS39, RAB51, RAB52, RAB7*, and *PEP12*, are known to vital for fungal development and plant infection (Li et al., 2017, 2019; Xie et al., 2016; Yang et al., 2020; Zheng et al., 2015). Previous studies have shown that *FgVAC1* is an essential gene in *S. cerevisiae* Σ1278b and *A. niger*, while deletion of this gene in *A. nidulans* and *C. albicans* results in severe growth defects (Abenza et al., 2012; Dowell et al., 2010; Franke et al., 2006; van Leeuwe et al., 2020). Similarly, our study underscores the essential role of FgVac1 in maintaining proper fungal growth and development. Phylogenetic analysis (Fig. 1) and the significant growth defects observed in the *P*_*ZEAR*_*-FgVAC1* mutant (Fig. 2) highlight the conserved and vital role of FgVac1 across various fungi. The use of the inducible promoter *P*_*ZEAR*_ allowed for controlled expression of *FgVAC1*, with the addition of β-estradiol effectively restoring gene expression and associated phenotypes.

Our colocalization analysis with FgRab51 revealed that FgVac1 is predominantly localized to early endosomes, exhibiting dynamic movements (Fig. 3, Videos S1 and S2). Previous studies with FgVps35 and FgVps39 also demonstrated their colocalization with FgRab51/FgRab5, indicating similar dynamic mobility. Inhibition of microtubules with a destabilizing agent hindered the dynamic mobility of these proteins (Li et al., 2017; Zheng et al., 2016), indicating the crucial role of microtubules in facilitating early endosomal movements.

CPY, a soluble protease, is transported from the late Golgi to the vacuole through endosomal compartments. When the CPY pathway is impaired, CPY is mis-sorted and can be diverted through the secretory pathway (Bonangelino et al., 2002). The proper localization of FgCpy1, a marker for vacuolar protein sorting (Bonangelino et al., 2002; Ohneda et al., 2005), was disrupted in the *P*_*ZEAR*_-*FgVAC1* mutant (Fig. 4). Unlike *C. albicans vac1* mutants, which exhibit vacuole fragmentation (Veses et al., 2009), such phenotypes were not prominent in *F. graminearum P*_*ZEAR*_-*FgVAC1* mutant.

Furthermore, the impact of FgVac1 on metal ion homeostasis and resistance mechanisms was demonstrated by the heightened sensitivity of the *P*_*ZEAR*_-*FgVAC1* mutant to zinc and iron, likely due to impaired vacuolar function (Fig. 5). Vacuoles play crucial roles in metal ion storage and detoxification, and the disruption of *FgVAC1* compromises these essential functions. This is consistent with findings in other fungi, where vacuolar dysfunction leads to metal ion sensitivity (Franke et al., 2006; Zhao et al., 2020). In *C. neoformans*, the SM protein Vps45 facilitates iron delivery to the vacuole and sense of iron repletion, thereby affecting iron homeostasis (Caza et al., 2018).

FgVac1 also plays a critical role for the production of DON, a key virulence factor in *F. graminearum*. Reduced DON production in the *P*_*ZEAR*_-*FgVAC1* mutant, coupled with downregulation of *TRI4* and *TRI5* genes, correlates with its diminished virulence potential (Fig. 6). The involvement of vesicle and vacuolar compartments in secondary metabolism pathways further emphasizes FgVac1's impact on mycotoxin biosynthesis and storage (Hong & Linz, 2008).

In summary, FgVac1 is involved in multiple developmental and pathogenic processes in *F. graminearum*, including vegetative differentiation, reproduction, and wheat infection. FgVac1 localized to early endosomes, and impairment of FgVac1 led to the mis-sorting of the vacuolar protein FgCpy1. Using a CPR approach, we confirmed that *FgVAC1* is an essential gene for fungal development. These findings underscore the significance of vacuolar protein sorting mechanisms in fungal physiology and pathogenicity. Further research efforts might aim to identify Golgi-derived cargoes specifically targeted by FgVac1, thereby enhancing our understanding of vacuolar protein sorting in fungal pathogens and offering potential targets for disease control strategies.

## Supplementary Information

Below is the link to the electronic supplementary material.

Video S1. Mobility of FgVac1-GFP

Video S2. Mobility of RFP-FgRab51

Supplementary file3 (PDF 222 KB)

## Data Availability

All data generated or analyzed in this study are included in the article or its supplementary information.
