# Supplementary material for: FgVAC1 is an Essential Gene Required for Golgi-to-Vacuole Transport and Fungal Development in Fusarium graminearum
Source: J Microbiol. 2024 Jul 30;62(8):649–60. doi: 10.1007/s12275-024-00160-x (PMC11379736; doi:10.1007/s12275-024-00160-x)
Supplement: Supplementary file 3 — Supplementary file3 (PDF 222 KB) [file 12275_2024_160_MOESM3_ESM.pdf]

Table S1. ***F. graminearum*** **strains used in this study**

**Strain**	**Genotype**	**Source**
Z-3639	*Fusarium graminearum* wild-type	Bowden & Leslie (1999)
P_{ZEAR}-*FgVAC1*	*HYG*-P_{ZEAR}-*FgVAC1*; Δ*Fgvac1*::*GEN*	This study
RFP-FgRab51	*HYG-RFP-FgRAB51*	This study
RFP-FgRab7	*HYG-RFP-FgRAB7*	This study
FgVac1-GFP::RFP-FgRab51	*HYG-FgVAC1-GFP*; *HYG-RFP-FgRAB51*	This study
FgVac1-GFP::RFP-FgRab7	*HYG-FgVAC1-GFP*; *HYG-RFP-FgRAB7*	This study
FgCpy1-GFP	*HYG-FgCPY1-GFP*	This study

Table S2. Primers used in this study

Purpose	Name	Sequence
qRT-PCR	CYP1-RT-F	TCAAGCTCAAGCACACCAAGAAGG
	CYP1-RT-R	GGTCCGCCGCTCCAGTCT
	FgVAC1-RT-F	GCTACGAGGCTTGGATGTGTTTG
	FgVAC1-RT-R	AATGGTGTCGCGTGATGAGTTC
	TRI4-RT-F	TCGAGGCACAACAGAAGGGTATCC
	TRI4-RT-R	AATGTCGGCCTTGGTGGTGTC
	TRI5-RT-F	GCCATTTTGGACCTTTCTGCTCATT
	TRI5-RT-R	GCCATAGAGAAGCCCCAACACAAT
***FgVAC1* deletion**	FgVAC1-5F	CATCCTCTACGCCTTTGTGACG
	FgVAC1-5N	CCATCGAGGGGACTTTGTTAGG
	FgVAC1-5R	gcacaggtacacttgtttagagGCTGCAACGCTGTGGGAAGT
	FgVAC1-3F	ccttcaatatcatcttctgtcgGACAAATCGGAGTTCTTCACAGGA
	FgVAC1-3N	GAAGGCCAAGCAAGCGTCTCT
	FgVAC1-3R	CGCACCCAACAGTCAAAATACAC
***P_{ZEAR}-FgVAC1* fusion**	PZEAR-F	tatagggcgaattgggtactcaaattggttCATGCCCTGGCGTTGAAGTT
	PZEAR-R	CATGGTTACTTTCGTTCTCTCTGGTC
	FgVAC1-F	gagagaacgaaagtaaccatgATGTCGGGCCGCAAGCTG
	FgVAC1-R	cgagaaactaggccagcagtagacacAGGGGGATAGGGTGGAATAGTTG
***FgVAC1-GFP* fusion**	FgVAC1-GFP-F	tttcgtaggaacccaatcttcaaaATGTCGGGCCGCAAGCTG
	FgVAC1-GFP-R	aacagctcctcgcccttgctcacgttaattaagcctccgcctccgcctccAGTGACACCATTGCGATATACACCC
***FgCPY1-GFP* fusion**	FgCPY1-GFP-F	tatagggcgaattgggtactcaaattggttCCCTGGTCCATGATGAGCACTTA
	FgCPY1-GFP-R	cccggtgaacagctcctcgcccttgctcacGAACCACTCACCACTGAGCCAG
***RFP-FgRAB51* fusion**	RFP-F	ATGGTGAGCAAGGGCGAGG
	RFP-R	CTTGTACAGCTCGTCCATGCCG
	RAB51-P-F	CTGGGTCCTCGAATCGTAAGTTG
	RAB51-P-R	cctcctcgcccttgctcaccatTGTCGCGGGATCGCTCG
	RAB51-P-N	tatagggcgaattgggtactcaaattggttTGGCAACATCGACTTCGGTAATAG
	RAB51-ORF-F	cggcatggacgagctgtacaagggaggcggaggcggaggcttaattaacATGGCCGATTCCACCAACG
	RAB51-ORF-R	GTGAGTGCGCGAAAGGAAATAGAT
	RAB51-ORF-N	cgagaaactaggccagcagtagacacTTGCTCTGACGCAAAGGCTAAC
***RFP-FgRAB*7 fusion**	RAB7-P-F	GCAAAGCAGCAAGACAGCGTATT
	RAB7-P-R	cctcctcgcccttgctcaccatGGTGAATAATCGTTTGTAAAAGGGG
	RAB7-P-N	tatagggcgaattgggtactcaaattggttCAAAGGTGGGGTTGATGTGGTT
	RAB7-ORF-F	cggcatggacgagctgtacaagggaggcggaggcggaggcttaattaacATGTCTTCTCGAAAGAAGGTTCTTC
	RAB7-ORF-N	cgagaaactaggccagcagtagacacTGCCTCGGACATGGACAGTTC
	RAB7-ORF-R	AGGTTCGCGGCTGCTGACTAC
